# On Chinoidine

**Published:** 1848-12

**Authors:** 


*On Chinoidine.* By J. S. Unzicker, M. D., of Cincinnati..—Chinoidine has been for several years past extensively used in Germany, but up to this time has not been generally known in this country.

It is a resinous substance, and was first discovered and separated from the mother liquor of sulph. quinine, by Sertuerner, a chemist. There are two kinds in commerce, the pure and impure. The genuine is hard, and has a smooth, glossy break, like the hepatic aloes, which it resembles in color. It is transported in oblong flat lumps weighing about one pound each, wrapped in wax paper; will dissolve perfectly in dilute sulphuric acid, alcohol and ether, forming a clear tincture of a brown color.

It is insoluble in cold, but scantily soluble in warm water, imparting to the latter a strongly bitter taste.

The adulterated comes in jars, and is soft; will only partly dissolve in alcohol and ether, not forming a clear tincture like the other

Chinoidine has been analyzed by a number of chemists, but as yet no satisfactory results obtained. It contains, according to Liebig, identically the same composition as quinine, but differs only in form, one being crystalline, the other not, and therefore he terms it the amorphous quinine. Chemical investigations, as far as they have extended, have thrown an interesting light upon the testimonies borne to the efficacy of chinoidine in the treatment of intermittent fevers; and there is no doubt in my mind, that the amorphous, if prescribed in its pure state, like the crystalline quinia, will produce the same effect on the animal organism, only that the effects of the former are more lasting.

I have used chinoidine for the last three years more extensively than quinine, and on an average with better success, especially in cases of long standing.

In a number of obstinate cases of long standing, who came here from the Wabash country, quinine was first given without any effect. I then gave the chinoidine, which not only checked the paroxysms, but no relapse followed afterwards.

From the experience which I had during the above time with this remedy, I think the return of the fever was one third less than when I used the quinine. It is particularly suited in cases where the digestive organs are not much debilitated. My mode of giving it is thus:—℞. Chinoidine. gr. xij.; rad. ipecac., gr. j.; morph. acet., gr. 1-4. Mix, and make into six pills, to be given in two doses, two hours previous to the chills. Some German physicians prefer to give the chinoidine in combination with cremor tartar and cloves, in the form of pills.

This I found would have the same effect as that of many grains of quinine with the same combination. After the chills were stopped, I would then give a teaspoonful of the tincture (ʒj. to ℥j. of alcohol) from two to three times daily in some claret wine, for several days afterwards.

It has been said by some, that the effects of chinoidine depended on the quantity of chinine and cinchonine it contained. This is an error; for suppose it did contain some chinine and cinchonine, how much could there be in twelve grains of chinoidine? Surely not half a grain of each! and would they have produced the effect above described? Certainly not. Many, as well as myself, have labored under this mistake, until they have learned to distinguish between the pure and impure article, for if it is pure the results will be alike.

Chinoidine will undoubtedly, in time, become the great remedy of practitioners in the West; for when we consider the high price of quinine, $3 per ounce, and chinoidine at 40 cents per ounce, and besides a more effectual remedy than the former, no doubt is left of its adoption into general use.— *Western Lancet.*

